# Source of MDR infections in an ICU: busting the myth

**DOI:** 10.1186/cc14186

**Published:** 2015-03-16

**Authors:** R Agrawal

**Affiliations:** 1 FEHI, New Delhi, India

## Introduction

MDR infections in the ICU are not nosocomial all the time, as perceived commonly. We performed a 2-year retrospective study to analyze the source of culture positivity in a medical ICU and to identify which types of infections are more prevalent.

## Methods

The data of a 35-bed medical ICU were analyzed from November 2012 to October 2014. The source of culture positivity was divided into three groups: patients admitted from the ER to the ICU who were referred from other hospitals or direct admissions, the second group was patients admitted within the hospital but outside the ICU for the first 48 hours, and the third group was ICU-acquired infections. We also analyzed the data for type of infections, whether Gram-negative, Gram-positive or fungal.

## Results

There were 1,051 cultures positive in a 2-year period. In total, 46.8% (*n *= 492) of cultures were already positive on admission, which denotes community-acquired and referred patients from other hospitals. A total of 31.1% (*n *= 327) of cultures were positive from patients admitted to general wards for more than 48 hours and then transferred to the ICU. Twenty-two percent (*n *= 232) of cultures were ICU-acquired infections. The data show community-acquired and hospital-acquired infections are the bulk of the culture load in an ICU. This could be attributed to increased surveillance and adherence to infection control practices in the ICU which may not be followed stringently in other parts of the hospital. Overuse of broad-spectrum antibiotics in community and primary care hospitals has resulted in a spurt in growth of resistant infections. This has reached an alarming level in developing countries. Out of total cultures positive 78.3% (*n *= 822) were Gram-negative infections which included community-based and non-ICU infections.

## Conclusion

Antibiotic stewardship and strict adherence to infection control protocols in hospitals and guidelines for general practitioners can significantly reduce the load of resistant organisms in the ICU. This may eventually improve patient outcomes and help in preserving the antibiotics for future generations.
